# The impact of different geometric assumption of mitral annulus on the assessment of mitral regurgitation volume by Doppler method

**DOI:** 10.1186/s12947-020-0187-6

**Published:** 2020-01-31

**Authors:** Wugang Wang, Zhibin Wang, Junfang Li, Kun Gong, Liang Zhao, Guozhang Tang, Xiuxiu Fu

**Affiliations:** Department of Echocardiography, The Affiliated Hospital of Qingdao University, No.16 Jiangsu Road, Qingdao, 266003 China

**Keywords:** Mitral regurgitation volume, Quantitative pulsed Doppler, Real time three-dimensional echocardiography, Mitral annulus

## Abstract

**Background:**

Mitral regurgitation volume (MRvol) by quantitative pulsed Doppler (QPD) method previously recommended suffers from geometric assumption error because of circular geometric assumption of mitral annulus (MA). Therefore, the aim of this study was to evaluate the impact of different geometric assumption of MA on the assessment of MRvol by two-dimensional transthoracic echocardiographic QPD method.

**Methods:**

This study included 88 patients with varying degrees of mitral regurgitation (MR). The MRvol was evaluated by QPD method using circular or ellipse geometric assumption of MA. MRvol derived from effective regurgitant orifice area by real time three-dimensional echocardiography (RT3DE) multiplied by MR velocity-time integral was used as reference method.

**Results:**

Assumption of a circular geometry of MA, QPD-MA_A4C_ and QPD-MA_PLAX_ overestimated the MRvol by a mean difference of 10.4 ml (*P* < 0.0001) and 22.5 ml (*P* < 0.0001) compared with RT3DE. Assumption of an ellipse geometry of MA, there was no significant difference of MRvol (mean difference = 1.7 ml, *P* = 0.0844) between the QPD-MA_A4C + A2C_ and the RT3DE.

**Conclusions:**

Assuming that the MA was circular geometry previously recommended, the MRvol by QPD-MA_A4C_ was overestimated compared with the reference method. However, assuming that the MA was ellipse geometry, the MRvol by the QPD-MA_A4C + A2C_ has no significant difference with the reference method.

## Introduction

Mitral regurgitation (MR) is one of the most common heart-valve disorder, and its prevalence increases with age [1]. In the clinical decision-making process regarding mitral valvular lesions, accurate determination of the severity of the MR is of major importance [2, 3]. Echocardiography is the first method of non-invasive assessment of MR, and mitral regurgitation volume (MRvol) is an important parameter to evaluate the severity of MR, which may be calculated by quantitative pulsed Doppler (QPD) method as previously recommended [4]. However, this method suffers from geometric limitations of two-dimensional (2D) echocardiography. In the QPD method, important geometric errors are made in calculating the cross-sectional area (CSA) of the mitral annulus (MA) because of the circular geometric assumption (the CSA of MA was derived as 0.785 d^2^, where d was the diameter of the MA in the apical four-chamber view) [5]. Recently, a series of studies have confirmed that MRvol using effective regurgitant orifice area (EROA) (direct planimetry of EROA by real time three-dimensional color Doppler echocardiography) multiplied by the MR velocity-time integral (VTI_MR_) was highly accurate [6–8]. Therefore, the aim of this study was to evaluate the impact of different geometric assumption of MA on the assessment of MRvol by the traditional 2D transthoracic echocardiographic (TTE) QPD method, by comparison with MRvol derived from EROA by real time three-dimensional color Doppler echocardiography.

## Methods

### Study population

This study included 88 patients (55 men, 33 women; mean age, 48.2 ± 14.0 years) with varying degrees of MR of different etiologies on color Doppler echocardiography between October 2011 and August 2017. The etiology of MR was ischemia in 32, idiopathic dilated cardiomyopathy in 26, mitral valve prolapse (MVP) in 30. MVP is diagnosed in the parasternal long-axis view as systolic displacement of the mitral leaflet into the left atrial of at least 2 mm from the MA plane [4]. Exclusion criteria included aortic regurgitation or stenosis, mitral stenosis, atrial fibrillation, frequent atrial or ventricular premature beats, congenital heart disease, hypertrophic cardiomyopathy, and poor general image quality. This study was approved by the institutional review board of The Affiliated Hospital of Qingdao University, and all patients underwent echocardiographic examination because of clinical indications and gave written informed consent.

### Echocardiographic examination

2D and real time three-dimensional (3D) echocardiography (RT3DE) were performed using the iE33 ultrasound system (Philips Healthcare, Amsterdam, The Netherlands).

### 2D TTE: MRvol by QPD method using different geometric assumption of MA

2D TTE was performed with a S5–1 transducer, and patients were imaged in the left lateral decubitus position. The MA diameter was measured between the inner edges of the base of posterior and anterior leaflets in early to mid diastole at maximal mitral valve (MV) opening in the apical four-chamber (A4C), apical two-chamber (A2C) and parasternal long-axis (PLAX) view [5, 9]. The left ventricular outflow tract (LVOT) diameter was measured just below the aortic valve in early to mid systole in the PLAX view [5, 9]. The pulsed Doppler sample was carefully placed as parallel as possible to the blood flow in the A4C and apical five-chamber (A5C) views to obtain the Doppler spectral profiles of the MA and LVOT. The sample volume was positioned at the level of the MA and LVOT. The modal velocity profile on Doppler recordings was traced to obtain the velocity-time integral (VTI) [5, 9]. Data from three cardiac cycles was averaged.

**Fig. 1:**
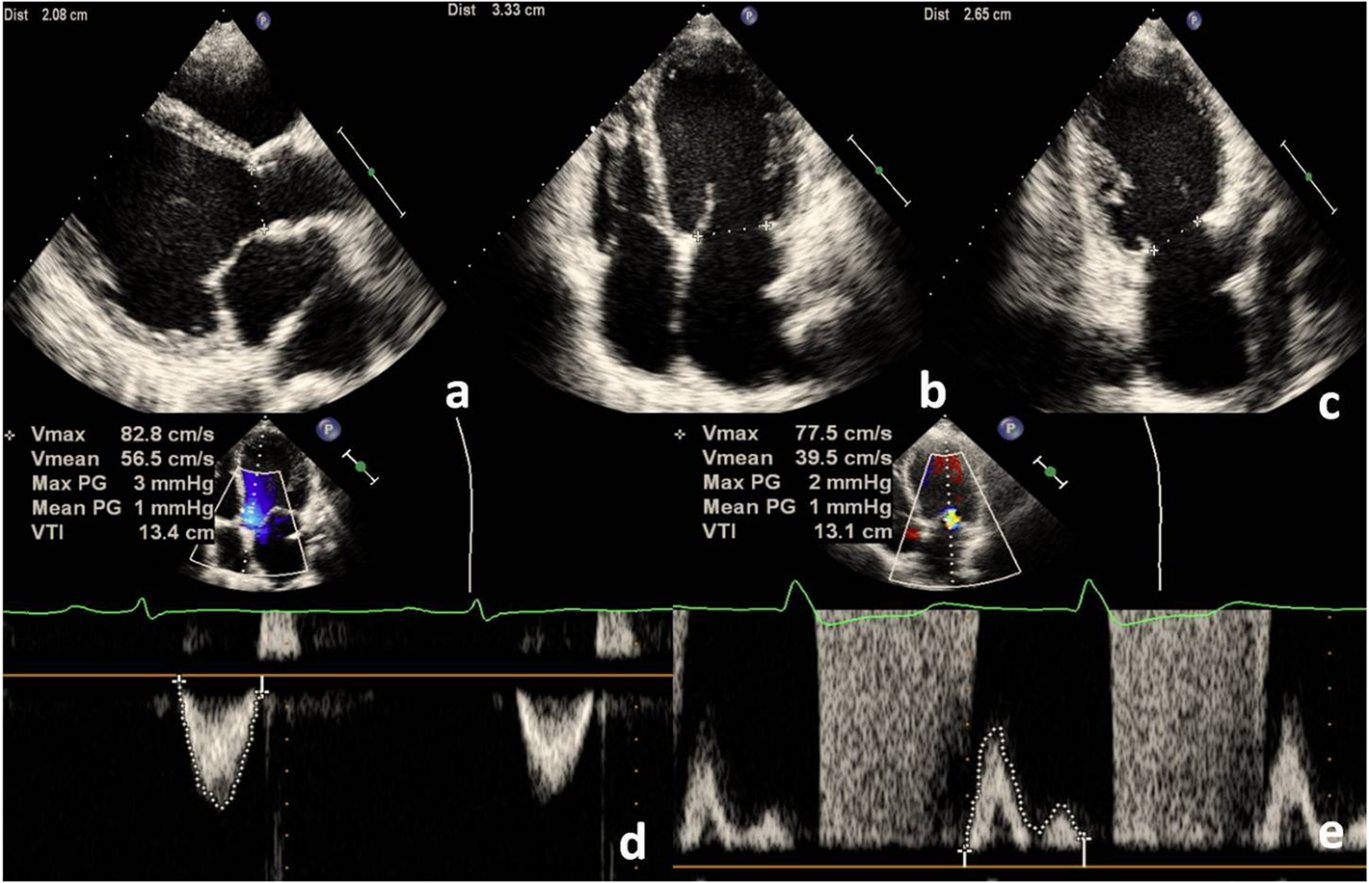
QPD calculations of MRvol assuming that the MA is ellipse geometry. **a** The diameter of LVOT was measured in the PLAX view, and the CSA_LVOT_ was derived as 0.785 d^2^. **d** LVOT pulsed Doppler was traced to obtain the VTI_LVOT_. Thus, SV_LVOT_ = 0.785 d^2^ × VTI_LVOT_ = 0.785 × 2.08^2^ × 13.4 = 45.51 ml. **b** and **c** The diameter of MA was measured in the A4C and A2C view. The CSA_MA_ was derived as 0.785 × a × b. **e** MA inflow pulsed Doppler was traced to obtain the VTI_MA._ Thus, SV_MA_ = 0.785 × a × b × VTI_MA_ = 0.785 × 3.33 × 2.65 × 13.1 = 90.75 ml. In this example of MR, MRvol = SV_MA_ - SV_LVOT_ = 90.75–45.51 = 45.24 ml

MRvol by QPD was calculated as the difference between MA forward stroke volume (SV_MA_) and LVOT forward stroke volume (SV_LVOT_) (Fig. 1), that was MRvol = SV_MA -_ SV_LVOT_. SV_LVOT_ was calculated as the VTI of LVOT (VTI_LVOT_) multiplied by the cross-sectional area (CSA) of LVOT (CSA_LVOT_). The LVOT is circular and the CSA_LVOT_ is derived as: πd^2^/4, that is 0.785 d^2^. Thus, SV_LVOT_ = 0.785 d^2^ × VTI_LVOT_, where d is the diameter of the LVOT in the PLAX view [4].

**Fig. 2:**
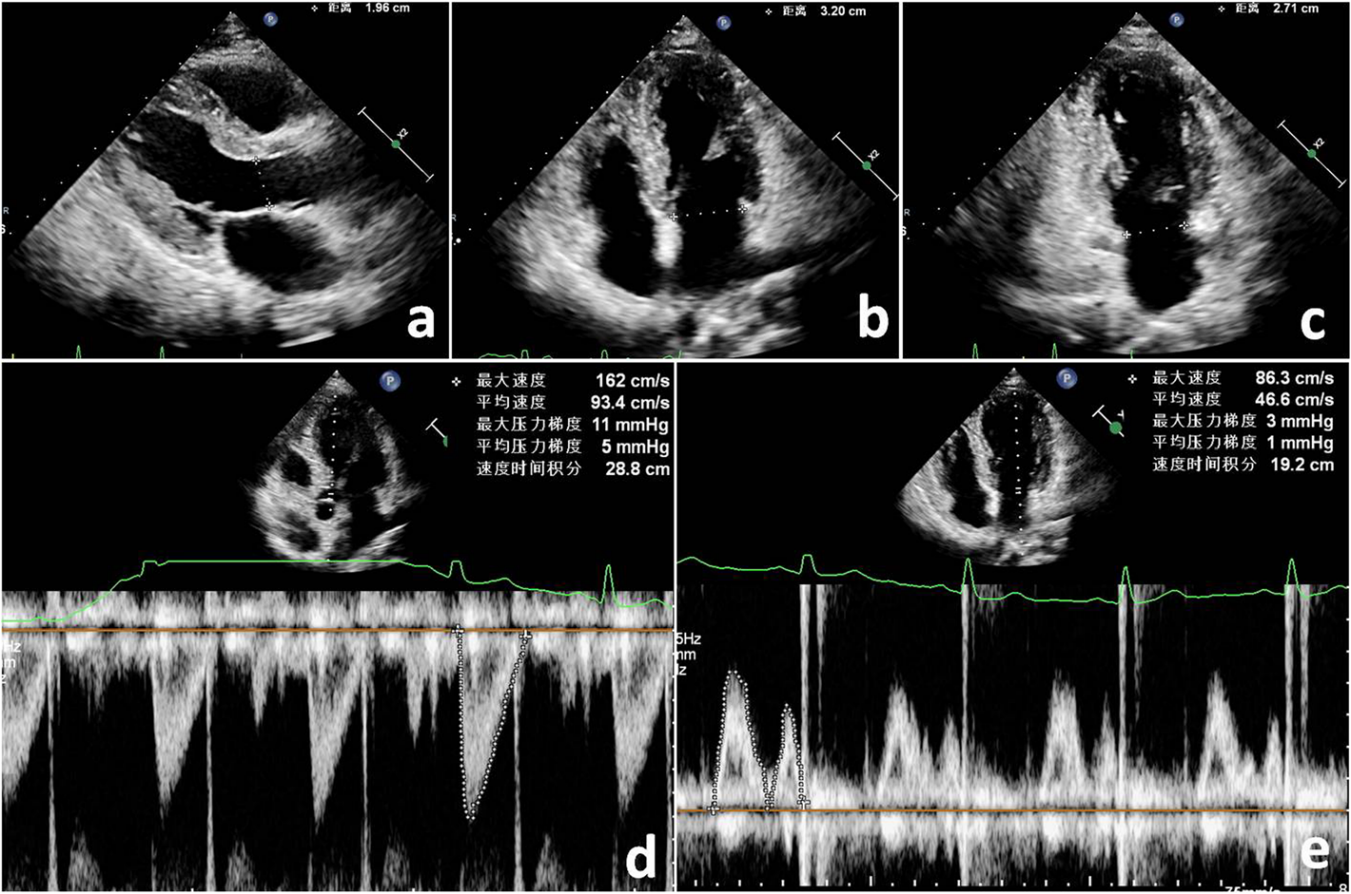
QPD calculations of MRvol assuming that the MA is circular geometry. **a** The diameter of LVOT was measured in the PLAX view, and the CSA_LVOT_ was derived as 0.785 d^2^. **d** LVOT pulsed Doppler was traced to obtain the VTI_LVOT_. Thus, SV_LVOT_ = 0.785 d^2^ × VTI_LVOT_ = 0.785 × 1.96^2^ × 28.8 = 86.85 ml. **b** and **c** The diameter of MA was measured in the A4C and A2C view. The CSA_A4C_ was derived as 0.785 × 3.2^2^, and CSA_A2C_ was derived as 0.785 × 2.71^2^. **e** MA inflow pulsed Doppler was traced to obtain the VTI_MA._ Thus, SV_A4C_ = 0.785 × 3.2^2^ × 19.2 = 154.33 ml, and SV_A2C_ = 0.785 × 2.71^2^ × 19.2 = 110.69 ml. In this example of MR, MRvol by QPD-MA_A4C_ = SV_A4C_ - SV_LVOT_ = 154.33–86.85 = 67.48 ml, and MRvol by QPD-MA_A2C_ = 110.69–86.85 = 23.84 ml

SV_MA_ was calculated as the VTI of MA (VTI_MA_) multiplied by the CSA of MA (CSA_MA_). Assuming that the MA is circular geometry (Fig. 2), the CSA_MA_ is derived as 0.785 d^2^. Thus, SV_MA_ = 0.785 d^2^ × VTI_MA_, where d was the diameter of the MA in the PLAX, A4C or A2C view. Assuming that the MA is ellipse geometry (Fig. 1), the CSA_MA_ is derived as 0.785 × a × b. Thus, SV_MA_ = 0.785× a × b × VTI_MA_, where a is the diameter of the MA in A4C and b in A2C view, a in A4C and b in PLAX view or a in A2C and b in PLAX view [4, 10, 11].

### Real-time 3D color Doppler echocardiograpy: MRvol by RT3DE

3D color Doppler data were acquired immediately after the 2D TTE using the same system equipped with a fully sampled matrix-array X3–1 transducer from the apical view, combining 7 small real-time subvolumes into a larger pyramidal volume. Nyquist limits were set between 40 and 60 cm/sec to avoid any overestimation or underestimation. Patients were asked to hold respiration during imaging acquisition.

**Fig. 3:**
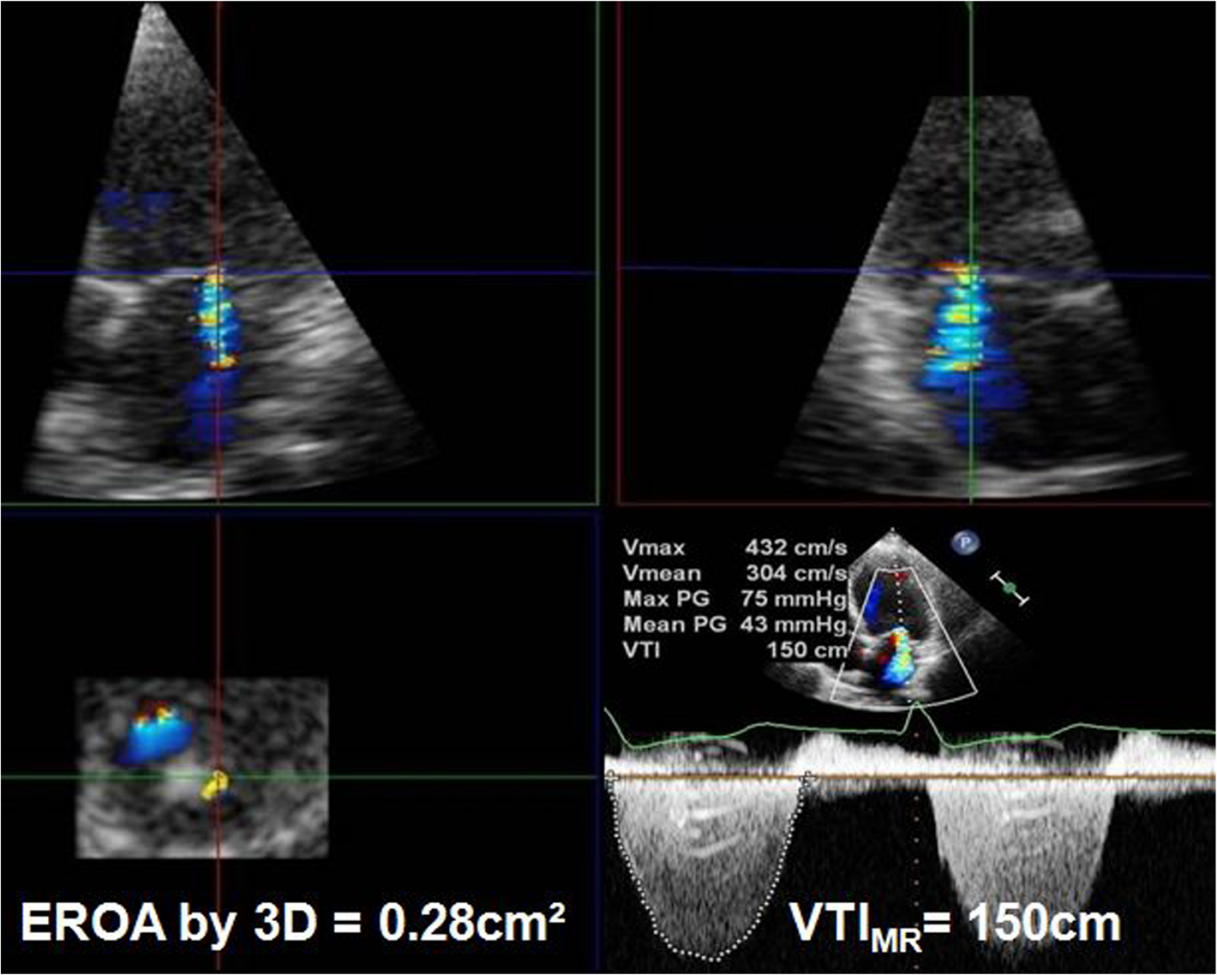
MRvol calculated as EROA by RT3DE multiplied by the VTI_MR_. The 3D color Doppler data was manually cropped by the cross-sectional plane perpendicularly to the regurgitant jet direction up to the smallest cross-sectional area of the regurgitant jet. The EROA was determined using manual planimetry of the color Doppler flow signal from an en face view. Example of a MR patient: EROA = 0.28 cm^2^, VTI_MR_ = 150 cm, MRvol = 0.28 × 150 = 42 mL

Three-dimensional color Doppler data sets were analyzed offline using software (QLAB version 7.1). Using multiplanar reconstruction of the 3D color Doppler data sets, a cross-sectional plane through the vena contracta perpendicular to the jet direction was selected, and the cross-sectional plane was then moved along the jet direction as far as the smallest cross-sectional area [7, 10]. The EROA was determined using manual planimetry of the color Doppler flow signal from an en face view, and the MRvol was calculated as EROA by RT3DE multiplied by the VTI_MR_ (Fig. 3). An MRvol > 60 ml was used to define severe MR [4].

### Statistical analysis

Continuous data were presented as mean ± SD. Categorical data were presented as percentages or absolute numbers. One factorial analysis of variance was used to compare the MA diameters measured in different views and the CSA_MA_ calculated using the different geometric assumption. Pearson’s correlation analysis was performed to evaluate the relation between QPD and RT3DE measurements of MRvol. Bland-Altman analysis was performed to evaluate the differences in MRvol assessed with QPD and RT3DE. Differences were considered statistically significant at *P* < 0.05. Statistical analysis was performed using MedCalc version 15.2 (MedCalc Software, Mariakerke, Belgium).

## Results

Clinical and echocardiographic characteristics of the MR patients are listed in Table 1.

**Table 1:** Patient characteristics (*n* = 88)

Variable	Value
Age (y)	48.2 ± 14.0
Men/women	55/33
Heart rate (beats/min)	74.8 ± 13.8
Systolic BP (mm Hg)	112.5 ± 18.7
Diastolic BP (mm Hg)	73.1 ± 9.5
LVEDD (cm)	5.9 ± 1.1
LVESD (cm)	4.5 ± 1.5
LVEF (%)	53.1 ± 18.5
LVOT diameter (mm)	2.0 ± 0.1
VTI_LVOT_ (cm)	17.3 ± 5.0
VTI_MA_ (cm)	17.9 ± 5.4
VTI_MR_ (cm)	131.1 ± 29.9
EROA by RT3DE (cm^2^)	0.33 ± 0.14

*BP* Blood pressure, *LVEDD* Left ventricular end-diastolic diameter, *LVESD* Left ventricular end-systolic diameter, *LVEF* Left ventricular ejection fraction, *EROA by RT3DE* The EROA was measured by manual planimetry of the 3D color Doppler signal; Data are expressed as mean ± SD or as number

### Comparison of MA diameter in different views and CSA_MA_ based on different geometric assumption

As listed in Table 2, ANOVA showed significant differences among the 2D TTE diameters of the MA in three different views. The MA diameters in PLAX view were larger than the MA diameters in A4C or A2C view (MA_PLAX_ vs MA_A4C_ or MA_A2C_: 3.0 ± 0.4 vs 2.9 ± 0.4 or 2.7 ± 0.3, *P*<0.001). As for CSA of the MA, the CSA_PLAX_ derived from circular geometric assumption was larger than CSA_A4C_ and CSA_A2C_ or CSA_PLAX + A4C_, CSA_PLAX + A2C_ and CSA_A4C + A2C_ derived from ellipse assumption (*P*<0.001 for all).

**Table 2:** Results of ANOVA analysis for MA diameter and CSA_MA_

Variable	Value	ANOVA analysis	
		*F*	*P*
MA diameter (cm)			
MA_PLAX_	3.0 ± 0.4	18.471	<0.001
MA_A4C_	2.9 ± 0.4		
MA_A2C_	2.7 ± 0.3		
Circular assumption (cm^2^)			
CSA_PLAX_	7.1 ± 1.9	9.992	<0.001
CSA_A4C_	6.6 ± 1.7		
CSA_A2C_	5.7 ± 1.2		
Ellipse assumption (cm^2^)			
CSA_PLAX + A4C_	6.8 ± 1.6		
CSA_PLAX + A2C_	6.3 ± 1.4		
CSA_A4C + A2C_	6.1 ± 1.3		

*MA*_*PLAX*_*, MA*_*A4C*_*and MA*_*A2C*_*diameter* The MA diameter was measured in PLAX, A4C and A2C view, *CSA*_*PLAX*_ The CSA of MA was calculated from the MA diameter in PLAX view using circular geometric assumption (CSA = 0.785 d^2^), *CSA*_*PLAX + A4C*_ The CSA of MA was calculated from the MA diameter in PLAX and A4C view using ellipse assumption (CSA = 0.785 × a × b)

### Assumption of a circular geometry of MA, MRvol by QPD compared with reference method

**Fig. 4:**
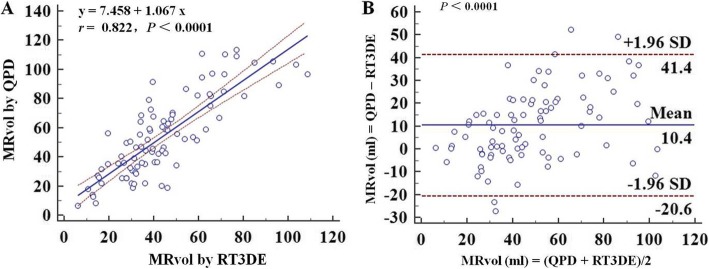
Assumption of a circular geometry of MA, linear regression plot and Bland-Altman plot showing correlations (**a**) and agreement (**b**) between MRvol by QPD-MA_A4C_ and RT3DE

**Fig. 5:**
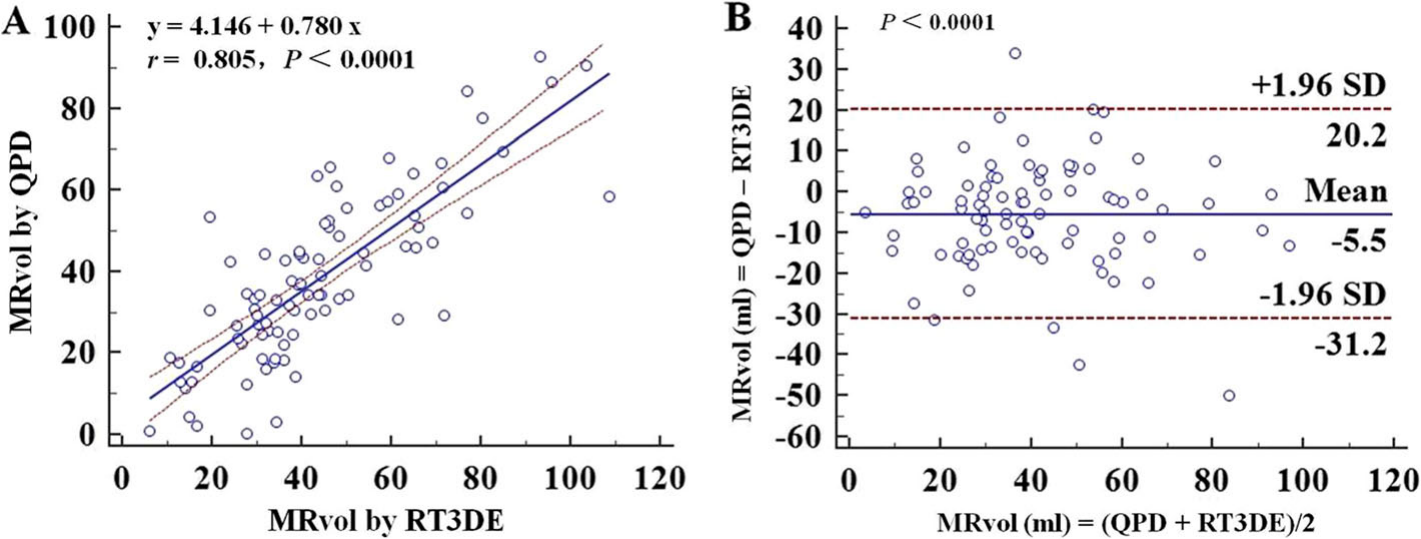
Assumption of a circular geometry of MA, linear regression plot and Bland-Altman plot showing correlations (**a**) and agreement (**b**) between MRvol by QPD-MA_A2C_ and RT3DE

Compared with MRvol by RT3DE, MRvol by QPD-MA_A4C_ and QPD-MA_A2C_ showed good correlation (*r* = 0.822, *P* < 0.0001; *r* = 0.805, *P* < 0.0001), while MRvol by QPD-MA_PLAX_ demonstrated poor correlation (*r* = 0.574, *P* < 0.0001). QPD-MA_PLAX_ and QPD-MA_A4C_ overestimated the MRvol by a mean difference of 22.5 ml (*P* < 0.0001) and 10.4 ml (*P* < 0.0001) compared with RT3DE (Fig. 4). However, QPD-MA_A2C_ underestimated the MRvol by a mean difference of 5.5 ml (*p* = 0.0002) compared with RT3DE (Fig. 5).

### Assumption of a ellipse geometry of MA, MRvol by QPD compared with reference method

**Fig. 6:**
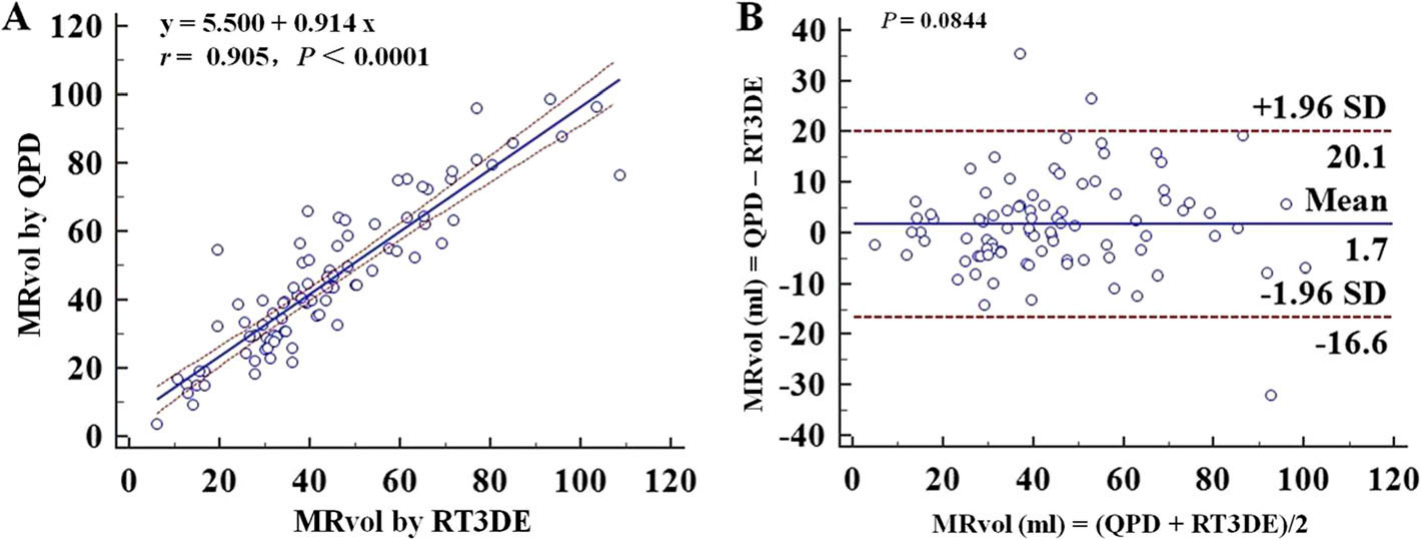
Assumption of an ellipse geometry of MA, linear regression plot and Bland-Altman plot showing correlations (**a**) and agreement (**b**) between MRvol by QPD-MA_A4C + A2C_ and RT3DE

MRvol by QPD-MA_PLAX + A4C_ and QPD-MA_PLAX + A2C_ demonstrated good correlation compared with RT3DE(*r* = 0.789, *P*<0.0001; *r* = 0.776, *P*<0.0001). QPD-MA_PLAX + A4C_ and QPD-MA_PLAX + A2C_ overestimated the MRvol by a mean difference of 15.2 ml (*P*<0.0001) and 6.8 ml (*P* = 0.0002) when compared with RT3DE. As for QPD-MA_A4C + A2C_, there was better correlation compared with RT3DE (*r* = 0.905, *P* < 0.0001), and the Bland-Altman analysis revealed no significant difference (mean difference = 1.7 ml, *P* = 0.0844) (Fig. 6). The correlation and difference between MRvol measured by QPD and RT3DE is summarized in Table 3.

**Table 3:** Results of Pearson’s correlation and Bland-Altman analysis for MRvol by QPD and reference methods

Method	MRvol (ml)	Pearson’s correlation analysis		Bland-Altman analysis		
		*r*	*p*	Mean difference (ml)	95% limits of agreement (ml)	*p*
Circular assumption						
QPD-MA_PLAX_	66.4 ± 41.1	0.574	<0.0001	22.5	− 43.6 ~ 88.6	<0.0001
QPD-MA_A4C_	54.3 ± 27.6	0.822	<0.0001	10.4	−20.6 ~ 41.4	<0.0001
QPD-MA_A2C_	38.4 ± 20.6	0.805	<0.0001	−5.5	−31.7 ~ 20.2	0.0002
RT3DE	43.9 ± 21.3					
Ellipse assumption						
QPD-MA_PLAX + A4C_	59.1 ± 29.5	0.789	<0.0001	15.2	−20.5 ~ 51.0	<0.0001
QPD-MA_PLAX + A2C_	50.6 ± 25.6	0.776	<0.0001	6.8	−24.9 ~ 38.5	0.0002
QPD-MA_A4C + A2C_	45.6 ± 21.5	0.905	<0.0001	1.7	−16.6 ~ 20.1	0.0844

*QPD-MA*_*PLAX*_ MRvol was measured by QPD using circular geometric assumption, and the MA diameter was measured in PLAX view, *QPD-MA*_*PLAX + A4C*_ MRvol was measured by QPD using ellipse assumption, and the MA diameter was measured in PLAX and A4C view, *RT3DE* MRvol was measured using EROA multiplied by the VTI_MR_

### Categorizations of MR severity according to different methods

On the basis of MRvol by RT3DE, 19 (21.6%) patients had severe MR (MRvol > 60 ml). Assuming that the MA is circular geometry, 42 (47.8%) patients had severe MR based on QPD-MA_PLAX_, 31 (35.2%) patients had severe MR based on QPD-MA_A4C_, and 12 (13.6%) patients had severe MR based on QPD-MA_A2C_. Assuming that the MA is ellipse geometry, 40 (45.4%) patients had severe MR based on QPD-MA_PLAX + A4C_, 28 (31.8%) patients had severe MR based on QPD-MA_PLAX + A2C_, and 22 (25%) patients had severe MR based on QPD-MA_A4C + A2C_.

Compared with MRvol by RT3DE, MR severity using QPD-MA_PLAX_, QPD-MA_A4C_, QPD-MA_PLAX + A4C_, and QPD-MA_PLAX + A2C_ were overestimated in 23 (26.1%) patients, in 12 (13.6%) patients, in 21 (23.8%) patients, and in 9 (10.2%) patients, respectively, and MR severity using QPD-MA_A2C_ was underestimated in 7 (10.2%) patients. Although MR severity using QPD-MA_A4C + A2C_ was overestimated in 3 (3.4%) patients, Chi-squared revealed no significant difference (*P* = 0.724).

## Discussion

The main finding of this study was that compared with the MRvol using EROA by RT3DE multiplied by the TVI_MR_, the MRvol was overestimated significantly by the 2D TTE QPD_A4C_ method previously recommended [2, 4]. The overestimates were caused by the circular geometric assumption of the MA, which led to the CSA _A4C_ and corresponding SV_MA_ being overestimated. In our study, assumption of an ellipse geometry of MA, MRvol calculated by QPD_A4C + A2C_ showed better correlation (*r* = 0.905, *P* < 0.0001) and had no significant difference (mean difference = 1.7 ml, *P* = 0.0844) with MRvol by RT3DE.

MRvol by QPD is simple in theory. Stroke volume (SV) at aortic valve or MV is derived as the product of CSA and VTI of flow at the LVOT or MA. In the absence of MR, SV determinations at LVOT and MA are equal. In the presence of MR, without any intracardiac shunt, the flow through MA is larger than through the LVOT. The difference between the two represents the MRvol [12]. For MRvol by QPD method, it is very important to accurately evaluate the CSA_MA_ and CSA_LVOT_. The calculation method of CSA_LVOT_ is nearly consistent (CSA_LVOT_ = πd^2^/4, where d was the diameter of the LVOT in the PLAX view) [2, 4, 9]. However, the calculation method of CSA_MA_ is controversial [5]. The MA diameter was measured in the A4C view and the CSA_MA_ was derived as 0.785 d^2^ (where d is the MA diameter in A4C) assuming that the MA is circular geometry as previously recommended [4, 9]. However, previous studies have been demonstrated that the MA has a saddle-shaped contour [13] and the CSA of the MA are oval, with the major and minor diameter [5, 14, 15]. Ren et al. [5] studied geometric errors of the MA by RT3DE. They found that the MA geometry was oval in the 3D en face views with a significant difference between the major and minor diameters. The 2D diameters of the MA_A4C_ was significantly different from both the major and minor diameters. Assuming that the MA was circular geometry, the CSA of the MA_A4C_ by 2D TTE overestimated the CSA compared with RT3DE [5]. In our study, the QPD-MA_PLAX_ and QPD-MA_A4C_ overestimated the MRvol (mean difference = 22.5 ml, *P* < 0.0001; mean difference = 10.4 ml, *P* < 0.0001) and QPD-MA_A2C_ underestimated the MRvol (mean difference = 5.5 ml, *P* = 0.0002) compared with the MRvol by RT3DE. A possible reason is that the 2D MA_PLAX_ and MA_A4C_ diameters may approach the 3D major diameters, and the MA_A2C_ may be close to the 3D minor diameters as previous findings [5, 16]. Based on the assumption of circular geometry, the monoplanar measurements of MA diameter and false geometric assumption are crucial factors of error using the 2D TTE QPD method. This error is important because the MA diameter is squared to derive the CSA_MA_ in the geometric circular assumption formula, which result in an overestimation of the CSA_PLAX_ and CSA_A4C_ and an underestimation of the CSA_A2C_. Because of these, the corresponding SV_MA_ calculated by CSA_PLAX_ or CSA_A4C_ is overestimated, and the corresponding SV_MA_ calculated by CSA_A2C_ is underestimated. Consequently, the QPD-MA_PLAX_ and QPD-MA_A4C_ overestimated the MRvol and QPD-MA_A2C_ underestimated the MRvol, which may cause over- or underestimation of MR severity. In our present study, compared with MRvol by RT3DE, MR severity using QPD-MA_PLAX_ and QPD-MA_A4C_ were overestimated in 23 (26.1%) patients and in 12 (13.6%) patients, respectively, and MR severity using QPD-MA_A2C_ was underestimated in 7 (10.2%) patients.

Based on that the MA is oval with the major and minor diameters previously demonstrated [5, 14, 15]. In this study, assuming that the MA was ellipse geometry, there was no significant difference of MRvol (mean difference = 1.7 ml, *P* = 0.0844) between the QPD-MA_A4C + A2C_ and the RT3DE. This is because the MA_A4C_ diameters may approach the 3D major diameters, and the MA_A2C_ may be close to the 3D minor diameters. The CSA_MA_ calculated by 2D MA_A2C_ and MA_A4C_ diameters using ellipse geometric assumption formula (CSA_MA_ = 0.785 × a × b) may be closer to the real CAS_MA_, which led to an accurate evaluation of corresponding SV_MA_ and MRvol by QPD-MA_A4C + A2C_ and may more accurately assess MR severity. In our present study, although MR severity using QPD-MA_A4C + A2C_ was slightly overestimated in 3 (3.4%) patients, Chi-squared revealed no significant difference (*P* = 0.724). Since the MA_PLAX_ diameter was larger than the MA_A4C_ (3.0 ± 0.4 vs 2.9 ± 0.4, *P* < 0.001), which resulted in the fact that the CSA_PLAX + A2C_ was larger than the CSA_A4C + A2C_, the corresponding MRvol by QPD-MA_PLAX + A2C_ was overestimated (mean difference = 6.8 ml, *P* = 0.0002) compared with the RT3DE. As for the overestimation of MRvol (mean difference = 15.2 ml, *P* < 0.0001) by QPD-MA_PLAX + A4C_, this could be related to the fact that the MA_A4C_ diameter was larger than the MA_A2C_ (2.9 ± 0.4 vs 2.7 ± 0.3, *P* < 0.001), which led to an overestimation of the corresponding CSA _PLAX + A4C_ and SV_MA_, thus overestimating the MRvol. The smaller difference of the result between the MRvol by QPD-MA_A4C + A2C_ and RT3DE was probably because the MA has an elliptic shape with a saddle-shaped 3D structure, and there are dynamic changes in its shape and position in different diseases during the cardiac cycle [17–20].

Previous study by Lewis JF observed a high correlation between thermodilution- derived stroke volume and Doppler-determined mitral inflow volume, and did not find significant difference between the use of assumption of a circular geometry of MA from the A4C view and the use of assumption of an ellipse geometry of MA from the A4C and A2C views [21]. However, the study by Rokey R found that the average regurgitant volume by the Doppler method (6.04 ± 3.09 l/min), assuming that the MA was circular geometry from the A4C view, was slightly higher than that obtained by angiography (5.33 ± 3.48 l/min), although not significant [22]. Similar results were obtained in study by Enriquez-Sarano M in which the assumption of a circular geometry of MA from the A4C view by the Doppler method mildly overestimated the MA stroke volume and significantly overestimated regurgitant volume [23]. Most of the earlier studies of Doppler method was mainly based on the standard angiographic grading method, which is subjective and influenced by many factors, including catheter position, rhythm disturbances, amount and velocity of dye injected, chamber size, and radiogram penetration [24]. Even the quantitative angiography, which makes use of left ventriculographic stroke volume for mitral valve flow and thermodilution for cardiac output, has conspicuous limitations. The error of cardiac output measurement is between 5 and 10% for thermodilution and between 10 and 15% for angiography, leading to a greater error when they are combined into the MR [24].

Echocardiography is the most commonly method for evaluating MR severity, and the QPD method has been successfully introduced into routine clinical practice for assessment of MR severity [4]. The QPD method is based on accurately evaluating the CSA_MA_ and CSA_LVOT_. The MA diameter was measured in the A4C view and the CSA_MA_ was derived as 0.785 d^2^ assuming that the MA is circular geometry as previously recommended [4]. Unfortunately, the human left heart and mitral valve do not provide the ideal conditions for the application of assumption of a circular geometry of MA, which eventually diminish the accuracy of assessment of MR severity. Previous studies have been demonstrated that the MA has a saddle-shaped contour and the CSA of the MA are oval [5, 13–15]. Therefore, our aim of the present study is to find the appropriate geometric model and the optimal MA diameters combination for traditional 2D TTE through systematic research, so as to measure the MRvol by 2D TTE QPD more accurately. To the best of our knowledge, no clinical studies have assessed the impact different geometric assumption of MA on the assessment of MRvol by QPD. This study showed that the QPD-MA_A4C_ overestimated the MRvol assuming that the MA was circular geometry as previously recommended, and assuming that the MA is ellipse geometry, the MRvol with QPD-MA_A4C + A2C_ correlated well and had good agreement compared with MRvol using EROA by RT3DE multiplied by the VTI_MR_. The QPD-MA_A4C + A2C_ provided more accurate assessment of MRvol using ellipse assumption of MA than the QPD-MA_A4C_ applying circular assumption previously recommended.

### Limitations

First, a limitation of a true gold standard for calculating MRvol was absent in the present study. In this study, the MRvol derived from EROA by RT3DE multiplied by the VTI_MR_ was used as the reference method, which has been documented as an accurate method [6, 7], and some studies have used it as reference method [10, 25]. However, RT3DE has limited spatial resolution of the reconstructed image, which may lead to biased results [26]. Second, this study did not evaluate the dynamic changes of 3D structure and CSA of the MA in different diseases and cardiac cycles. Third, in this study, the relationship between 3D MA diameters and 2D diameters in different views, as well as the geometry of LVOT were not evaluated, which may add to further errors in calculating MRvol by QPD. Fourth, this present study did not address the significance to stratify the results in primary and secondary MR, and further researches and follow-up data are necessary to explore. Finally, further studies are needed to confirm the results of MRvol by QPD-MA_A4C + A2C_ based on ellipse assumption of MA.

## Conclusion

Assuming that the MA is circular geometry as previously recommended, the MRvol by 2D TTE QPD-MA_A4C_ was overestimated compared with the MRvol derived from EROA by RT3DE multiplied by the VTI_MR_. However, assuming that the MA was ellipse geometry, the MRvol by the 2D TTE QPD-MA_A4C + A2C_ was accurate compared with the reference method.

## Data Availability

The data and materials used in this study are available from the corresponding author or the first author on reasonable request.

## Abbreviations

2D: Two-dimensional
3D: Three-dimensional
A2C: Apical two-chamber
A4C: Apical four-chamber
A5C: Apical five-chamber
CSA: Cross-sectional area
CSA_A2C_: The CSA of MA was calculated from the MA diameter in A2C view using circular geometric assumption
CSA_A4C_: The CSA of MA was calculated from the MA diameter in A4C view using circular geometric assumption
CSA_LVOT_: CSA of LVOT
CSA_MA_: CSA of MA
CSA_PLAX + A2C_: The CSA of MA was calculated from the MA diameter in PLAX and A2C view using ellipse assumption
CSAP_LAX + A4C_: The CSA of MA was calculated from the MA diameter in PLAX and A4C view using ellipse assumption
CSA_PLAX_: The CSA of MA was calculated from the MA diameter in PLAX view using circular geometric assumption
EROA: Effective regurgitant orifice area
LVOT: Left ventricular outflow tract
MA: Mitral annulus
MA_A2C_: The diameter of MA was measured in the A2C view
MA_A4C_: The diameter of MA was measured in the A4C view
MA_PLAX_: The diameter of MA was measured in the PLAX view
MR: Mitral regurgitation
MRvol: Mitral regurgitation volume
MV: Mitral valve
MVP: Mitral valve prolapse
PLAX: Parasternal long-axis
QPD: Quantitative pulsed Doppler
QPD-MA_A2C_: MRvol was measured by QPD using circular geometric assumption, and the MA diameter was measured in A2C view
QPD-MA_A4C_: MRvol was measured by QPD using circular geometric assumption, and the MA diameter was measured in A4C view
QPD-MA_A4C+A2C_: MRvol was measured by QPD using ellipse assumption, and the MA diameter was measured in A4C and A2C view
QPD-MA_PLAX_: MRvol was measured by QPD using circular geometric assumption, and the MA diameter was measured in PLAX view
QPD-MA_PLAX+A2C_: MRvol was measured by QPD using ellipse assumption, and the MA diameter was measured in PLAX and A2C view
QPD-MA_PLAX+A4C_: MRvol was measured by QPD using ellipse assumption, and the MA diameter was measured in PLAX and A4C view
RT3DE: Real time three-dimensional echocardiography
SV: Stroke volume
SV_LVOT_: LVOT forward stroke volume
SV_MA_: MA forward stroke volume
TTE: Transthoracic echocardiographic
VTI: Velocity-time integral
VTI_LVOT_: VTI of LVOT
VTI_MA_: VTI of MA
VTI_MR_: MR velocity-time integral
